# Standardisation of inactivated influenza vaccines—Learning from history

**DOI:** 10.1111/irv.12543

**Published:** 2018-02-02

**Authors:** John M. Wood, Jerry P. Weir

**Affiliations:** ^1^ Formerly National Institute for Biological Standards and Control Potters Bar, Bushey Herts UK; ^2^ Division of Viral Products Center for Biologics Evaluations and Research, Food and Drug Administration Silver Spring MD USA

**Keywords:** influenza vaccine, potency, SRID

## Abstract

The single radial immunodiffusion assay has been the accepted method for determining the potency of inactivated influenza vaccines since 1978. The worldwide adoption of this assay for vaccine standardisation was facilitated through collaborative studies that demonstrated a high level of reproducibility and its applicability to the different types of influenza vaccine being produced at that time. Clinical evidence indicated the relevance of SRID as a potency assay. Unique features of the SRID assay are likely responsible for its longevity even as newer technologies for vaccine characterisation have been developed and refined. Nevertheless, there are significant limitations to the SRID assay that indicate the need for improvement, and there has been a substantial amount of work undertaken in recent years to develop and evaluate alternative potency assays, including collaborative studies involving research laboratories, regulatory agencies and vaccine manufacturers. Here, we provide an overview of the history of inactivated influenza vaccine potency testing, the current state of alternative assay development and the some of the major challenges to be overcome before implementation of new assays for potency determination.

## 1 EARLY DAYS OF INFLUENZA VACCINES

Inactivated influenza vaccines were first developed in the late 1930s from virus grown in either mouse lungs or chick embryos, and after a series of disappointing clinical trials, it was realised that the vaccines were too weak to stimulate a consistently robust immune response. After Hirst et al^1^ had demonstrated that higher vaccine doses could induce higher levels of serum antibody and that vaccine dose could be measured by an in vitro assay based on agglutination of chicken erythrocytes,^2^ the scene was set for more effective and consistent vaccine production. The in vitro assay was further standardised by the development of the chick cell agglutination (CCA) assay^3^ and the use of an International Standard for haemagglutination,^4^ but still there were problems. From international collaborative studies, the results of CCA assays were seen to vary between laboratories by up to twofold^5^ and with the advent of split virus and subunit vaccines, the CCA assay proved to be unreliable and not a good indicator of immunogenicity in humans. This was dramatically demonstrated during the “swine flu” A/New Jersey/76 (H1N1) vaccine trials in 1976, where the CCA values of newly developed split vaccines did not correlate with immunogenicity.^6^, ^7^

## 2 TWO NEW ASSAYS WERE DEVELOPED

It was fortuitous that a few years earlier, two new assays for influenza vaccine potency had been developed: one a single radial immunodiffusion (SRID) assay^8^ and the other a rocket immuno‐electrophoresis (IEP) assay.^9^ The SRID assay measured the concentration of haemagglutinin (HA) in influenza vaccines by virtue of its reaction with specific antibody to produce precipitin rings in an agarose gel, whereas the IEP assay utilised an electrophoretic current to elongate the precipitin rings into rocket‐shaped peaks. When SRID assays were used to test the “swine flu” vaccines in 1976, there was an excellent correlation between antigen content and vaccine immunogenicity, irrespective of whether the vaccine was whole virus, split virus or subunit.^6^, ^7^ However, this was not the case for the IEP assay, but more of that later. Thus, the foundations were laid for a significant change in the way that influenza vaccines were standardised. Schild and his colleagues were able to produce very potent antisera to purified HA in goats and rabbits and demonstrated that the antisera reacted well with HA released from detergent‐disrupted influenza virus in agarose gels.^8^, ^10^ They also worked out some of the key parameters of the assay including the influence of antigen and antiserum concentration, antigenic specificity of the assay and within‐laboratory reproducibility.^10^

## 3 SRID BECAME ACCEPTED

As SRID and IEP were new techniques, it was important that other laboratories acquired the technologies and that the results from different laboratories were in agreement. In August 1978, a World Health Organization (WHO) workshop was organised at the University of Bergen, Norway, by the National Institute for Biological Standards and Control (NIBSC), UK, and the Center for Biologics Evaluation and Research (CBER), USA. Twenty participants from the vaccine industry and regulatory agencies went “back to school” and learned how to run both assays. After this, the next stage was to investigate assay reproducibility in different laboratories. An international collaborative study to compare SRID and IEP was organised on behalf of WHO during the late 1970s,^11^ and 25 participants were asked to assay several influenza vaccines using supplied protocols. Results of the collaborative study showed only small differences in SRID results from different laboratories (Geometric Coefficient of Variation from 4% to 6%), and although there was good agreement between SRID and IEP for assay of whole virus vaccines, the IEP results for split vaccines were inconsistent. Such inconsistencies resulted in the IEP assay being rejected and only the SRID assay being considered for further development. The high level of reproducibility and the increasing clinical evidence^12^ of the relevance of SRID assays led to its acceptance for influenza vaccine standardisation by the WHO in 1978.^13^

## 4 SRID ASSAYS FOR VETERINARY INFLUENZA VACCINES

Influenza vaccines are also used in horses, pigs and chickens, although their use is considerably more restricted than that in humans. Approaches to vaccine standardisation in the veterinary world are also more localised and are led by market demands. Although SRID assays and matched reagents have been developed for equine (H7N7; H3N8),^14^ porcine (H1N1; H3N2) and chicken (H5N3)^15^ influenza vaccines, their use is neither widespread nor consistently applied. However, evidence for the clinical relevance of SRID assays has been reinforced by studies in horses^16^ and chickens,^15^ where the levels of protection clearly correlated to vaccine HA concentration measured by SRID.

## 5 KEY FEATURES OF THE SRID ASSAY

There are several unique features of the SRID assay that account for its worldwide adoption and longevity as the standard potency assay for inactivated influenza vaccines even as newer technologies for vaccine characterisation have evolved. Many of these features have been described previously,^17^ but are summarised briefly below.

### 5.1 Simplicity

The SRID assay is “low tech” and can be used in all manner of laboratories. At its simplest, it only needs glass plates, plastic moulds, agarose, boiling water bath, a supply of reagents (e.g standard antigen and strain‐specific antibodies) and a calibrated micrometre measuring device, although more sophisticated equipment such as image analysers are often used. This ensures that the assay is very robust and can be used in any laboratory, irrespective of their resources.

### 5.2 A functional assay

The SRID assay measures the quantity of HA by means of its reaction with specific antibody and has been shown to correlate with vaccine immunogenicity, first in the H1N1 vaccine trials during the late 1970s^6^, ^7^, ^12^ and in numerous vaccine clinical trials since then.

### 5.3 The need for a standard

An SRID assay typically compares a dilution series of a test antigen (e.g a vaccine) and a standard antigen, which has been calibrated in HA content. The HA content of the test antigen can thus be expressed in microgram of HA antigen activity, and in many parts of the world, the potency of seasonal influenza vaccines is 15 μg HA per strain per dose.^18^, ^19^

### 5.4 Antigenic specificity

The antisera used in SRID assays often cross‐react with closely related virus strains within a subtype, but do not cross‐react between subtypes. This means that SRID assays can be used to assay each component of a trivalent vaccine without any interference. The recent development of quadrivalent vaccines containing antigens for the two lineages of influenza B has been a somewhat more challenging issue for SRID testing^20^ because there is some degree of cross‐reactivity between antibodies to the two lineages. It is nevertheless important that the standard antigen and the vaccine are antigenically homologous.

### 5.5 Assay reproducibility

Since the first evaluation of SRID assay reproducibility in 1979, there have been several more such studies within the EU. In 1997, the SRID assay was accepted for influenza vaccine potency testing in the EU by the EU Committee for Medicinal Products for Human Use (CHMP)^18^ and it was desirable to maintain proficiency of EU laboratories by regular evaluation. These studies indicated that where the same protocols were used in each laboratory, interlaboratory variability was less than 10%, but where local methods were used, variability could range from 7% to 147%. The greater variability of local methods was largely due to laboratories not taking sufficient care to derive valid assays and when assays were improved, so too did reproducibility (Wood, European Directorate for the Quality of Medicine, unpublished; 1989, 1990, 1998, 2003).

### 5.6 Need for calibrated SRID reagents

Each time a new influenza strain is included in a vaccine, new SRID reagents must be produced. The process of reagent development and calibration has been much refined over the years so that it is now a rapid and effective collaboration between four WHO Essential Regulatory Laboratories (ERL) (CBER; National Institute of Infectious Diseases, Japan; NIBSC, UK; Therapeutic Goods Administration, Australia), each of which produce SRID reagents. Antisera are usually produced by hyperimmunising sheep with purified HA, whereas inactivated whole virus preparations, freeze‐dried to ensure stability, are used as antigen reagents. These preparations are calibrated to estimate the HA content by a collaborative process of the four laboratories, which involves protein and quantitative SDS‐polyacrylamide gel electrophoresis (SDS‐PAGE) analyses. There have been significant recent improvements to reduce ambiguities in the SDS‐PAGE analysis by incorporation of an earlier deglycosylation step.^21^ Calibration of SRID reagents is described in a protocol endorsed by the WHO Expert Committee on Biological Standardization.^22^

The need for specific SRID reagents is one advantage of the test as it ensures that reagents are matched precisely to each vaccine strain, but it can also be a disadvantage as it takes approximately 6‐8 weeks to make reagents available to vaccine manufacturers. Vaccine manufacturers have very little time to produce vaccine each season, and SRID reagents are needed before vaccines can be potency tested and formulated. Although reagent preparation rarely delays vaccine availability, it is always challenging to produce reagents in time. In recent years, there has been an increase in the number of vaccine strains being used by manufacturers, which has exacerbated the concern about reagent availability.

## 6 PANDEMIC CONSIDERATIONS

During the early stages of a pandemic, the timing of vaccine production and potency testing reagents development is even more critical. Furthermore, it is likely that a huge number of doses of pandemic vaccine will be needed and thus more reagents than normal will be needed to test the vaccines. Nearly all of our experience in preparing SRID reagents has been derived with seasonal vaccines, but more recent experience in preparing and testing candidate H5N1 vaccines and 2009 H1N1 pandemic vaccines has illustrated that new challenges could be faced in developing pandemic SRID reagents. For example, calibration of some H5N1 reagents was problematic due to inconsistencies in the SDS‐PAGE analysis, and also there were initial difficulties in the purification of HA from the H1N1pdm09 virus that was used to immunise sheep. Such difficulties impeded reagent development, which meant that some vaccine manufacturers had to use alternative physico‐chemical assays to determine potency of the initial lots of vaccine. Therefore, there are concerns that delays in SRID reagent availability could delay the availability of pandemic vaccines.

One of the major rate‐limiting steps in SRID reagent production is the preparation of antisera, but this could be overcome by utilising the well‐recognised cross‐reactivity of SRID antisera so that in the first stages of a pandemic, there could be an evaluation of existing SRID antiserum cross‐reactivity with the new pandemic virus HA. The feasibility of stockpiling SRID antisera in preparation for a pandemic has recently been suggested,^23^ and some progress in this direction has already been made.^24^ Another possibility to improve reliability of antiserum production is to use recombinant HA derived from suitable viral or bacterial vectors.^25^

## 7 NEW ASSAY DEVELOPMENT

### 7.1 The need to improve vaccine standardisation

In spite of the advantageous features of the SRID method that have contributed to its continual use as the traditional potency assay for nearly 40 years, there are some significant limitations to the assay that indicate the need for improvement and the development of alternative potency assays.^17^ In addition to the challenge of timely reagent preparation noted above, other limitations of the SRID include a relatively narrow dynamic range and questionable applicability to some newer types of influenza vaccines being developed.

In April 2014, the European Medicines Agency (EMA) provided guidelines for pandemic influenza vaccines, encouraging the development of alternative methods for antigen standardisation to bridge the phase when no such reagents are available.^26^

In July 2010, an important meeting jointly organised by Health Canada, the United States Food and Drug Administration (FDA), and the WHO was held in Ottawa, Canada, to assess the lessons learned from potency testing of 2009 H1N1 pandemic vaccines.^27^ Some of the key conclusions were as follows:

1 As it is likely that SRID will remain the primary potency assay for inactivated influenza vaccines for the foreseeable future, efforts should be made to improve the assay, including:
  - Harmonisation of assay method and reagent availability.
  - Improvement of the reagent calibration process.
2 To address some of the limitations of the SRID assay, alternative methods to determine vaccine potency should be developed and evaluated:
  - New assays should be low cost, not labour‐intensive, high throughput, high specificity, stability‐indicating, indicative of antigenic structure and vaccine effectiveness. Most of these are features of the SRID assay.
  - Development should include application of assays for newer vaccines, for example cell culture‐derived vaccines; recombinant vaccines; adjuvanted vaccines; virus‐like particles.
  - Validation of new assays could include bridging studies to SRID assays, animal studies and clinical trials.

### 7.2 Recent progress in the development of alternative influenza vaccine potency assays

A substantial amount of work has been undertaken, both before and since the Ottawa meeting, to develop and evaluate the feasibility of alternative methods for potency determination. Progress has been reviewed in two follow‐up workshops in July 2013 and January 2016 in London, UK, sponsored by the International Federation of Pharmaceutical Manufacturers & Associations (IFPMA), NIBSC, and the U.S. Department of Health and Human Services (HHS). In addition, two large collaborative studies, organised by these same groups, were conducted in 2015/2016 and 2017 to compare several of the methods for their ability to measure the quantity of influenza H1, H3 and influenza B HA in monovalent and multivalent vaccine samples provided by several manufacturers and to make a preliminary assessment of whether the techniques were able to distinguish denatured vaccine (e.g by heat stress) from untreated samples. Although these studies were not designed to rank assays or to determine a single best replacement assay, the data indicated the general feasibility of several methods and suggested that further development of alternative potency assays was warranted.

Any new influenza potency assay, whether developed for a new type of vaccine or as an SRID replacement assay for an existing vaccine, would need to measure a property of the vaccine antigen that predicts its clinical effect. The SRID meets this requirement using strain‐specific antibodies to measure conformationally correct HA antigen. It might be possible to bridge an alternative potency assay to SRID, but it may be necessary to correlate new alternative assays directly to immunogenicity and/or clinical benefit as has been suggested by others.^28^ Several of the methods currently under development as possible alternative influenza potency assays are briefly described below.

### 7.3 SDS‐PAGE combined with densitometry

One of the simplest techniques that have been explored as an influenza potency assay is the use of SDS‐PAGE combined with densitometry to quantify the HA content in vaccine samples.^29^ During the 2009 H1N1 pandemic, this assay was used to formulate vaccine for clinical trials and expedite the approval of an H1N1 vaccine in China. While useful in an emergency setting, quantification of HA by SDS‐PAGE and densitometry does not easily distinguish HA subtypes in a multivalent vaccine, is not expected to be stability‐indicating and is therefore most likely suitable for in‐process testing and interim analysis of reagents.

### 7.4 Reverse‐phase high‐performance liquid chromatography

Reverse‐phase high‐performance liquid chromatography (RP‐HPLC) is another commonly used technique for quantifying HA,^30^, ^31^ particularly during manufacturing in‐process testing. The methodology is fairly common, rapid and accurate and was used to formulate vaccine for some H1N1pdm09 clinical trials in the United States in 2009, although it was not used for vaccine approval or release. HA results determined by RP‐HPLC often correlate with those determined by SRID, but as with many alternative assays, the results are not always equivalent. If used as a stand‐alone method, RP‐HPLC is not capable of distinguishing denatured or stressed vaccine from unstressed vaccine. However, by introduction of a trypsin pre‐treatment step that preferentially digests HA that has been altered by stress conditions such as pH or heat, RP‐HPLC quantification becomes conformationally selective and a potential stability‐indicating potency assay.^32^

### 7.5 Mass spectrometry

Mass spectrometry (MS) techniques are able to accurately quantify HA in vaccines,^33^, ^34^, ^35^ and the isotope‐dilution MS (IDMS) method is able to quantify the HA subtypes in a multivalent vaccine, and using common tryptic peptides is not dependent upon strain‐specific standards.^34^, ^35^ Despite these notable advantages, IDMS is not stability‐indicating and the technique must be coupled with other methods to measure only conformationally correct HA. One such adaptation uses antibody immunocapture followed by IDMS quantitation as a potential vaccine potency assay.^36^, ^37^ This method is one of the few assays being developed that is not dependent upon calibrated reference antigens, but it does require appropriately characterised antibodies as described in more detail below.

### 7.6 Surface plasmon resonance

Several years ago, a surface plasmon resonance (SPR) technique that used recombinant HA (rHA), bound to the SPR biosensor, to compete with a mixture of HA antibody and HA antigen in solution was described as a possible method for quantifying vaccine HA.^38^ While this methodology appeared sensitive, its use would require both specific antibody and rHA reagents and further development of this assay approach has not been reported.

### 7.7 Antibody‐based assays

Antibody‐based assays are an attractive alternative to SRID because like SRID, they are capable of measuring a native conformation of the HA antigen. Indeed, a monoclonal antibody (mAb) ELISA was proposed as an SRID alternative more than 30 years ago,^39^ but only in the last few years have detailed studies been performed to try to define the best assay set‐up and identify the characteristics of antibodies best suited for potency measurement. Universal antibodies that recognise all influenza subtypes have been described and used in an ELISA format to quantify HA, but these antibodies cannot distinguish influenza subtypes and may not be stability‐indicating because they bind HA under denaturing conditions.^40^ In other studies, strain‐specific mAbs have been used to quantify HA in capture ELISAs^20^, ^41^, ^42^; these assays are able to quantify each HA subtype antigen in multivalent vaccine formulations and are able to detect loss of potency in vaccine samples subjected to stress conditions. Another antibody‐binding platform under development as a possible potency assay uses panels of mAbs printed onto a slide format.^43^, ^44^ Advantages to this methodology include the miniaturised set‐up, which utilises small amounts of reagents and samples, and the multiplex format, which allows the measurement of all components of a multivalent vaccine in the same assay with high throughput. Issues that remain to be resolved for all types of antibody capture‐based assays include defining the criteria for mAb selection and determining whether mAbs can be generated or made available during the short timeframe of either seasonal or pandemic vaccine production.

### 7.8 Receptor‐binding assays

Binding of influenza HA to its sialic acid receptor is an intrinsic property of a correctly folded trimeric HA molecule. To take advantage of this property, receptor‐binding alternative potency assays have been developed in ELISA^45^ and SPR^46^ formats. The advantages of a receptor‐binding approach are that it is selective for conformationally correct antigen and does not require strain‐specific antibody reagents for the assay set‐up. However, receptor binding does not discriminate antigen subtypes, so additional steps, such as subtype‐specific antibodies, are needed for analysis of multivalent vaccine samples.^45^

## 8 NEXT STEPS AND CHALLENGES FOR THE FUTURE

As the examples above hopefully demonstrate, remarkable progress has been made in development and evaluation of new influenza vaccine potency assays that may address some of the shortcomings of the current SRID assay. Nevertheless, major challenges will need to be overcome before a new method(s) for potency testing is implemented. There are now more types of influenza vaccines than ever. Cell‐based vaccines, recombinant protein vaccines and adjuvanted vaccines have all been licensed in recent years,^47^ and new types of influenza vaccines such as virus‐like particles and vectored vaccines are on the horizon. Some new vaccines may contain influenza antigens other than HA, for example neuraminidase (NA). Additional antigens such as NA that contribute to vaccine effectiveness will require their own assays for potency.^48^ In short, it may be unrealistic to expect one method to be the most appropriate assay for all types of inactivated influenza vaccines, and in fact, recent experience has indicated that SRID is unlikely to be a suitable assay for all of the various types of influenza vaccines being developed.

The current system of producing reagents for assay standardisation evolved along with SRID development at the time when all influenza vaccines were similar, either whole‐inactivated or split vaccine, as noted earlier. However, these reagents may not be suitable for assays that measure properties of the vaccine antigen that are different from those measured by SRID. It has been suggested by several groups that one of the possible reasons why there is often correlation, but not always equivalence, between SRID potency results and potency values determined by alternative methods is that the current SRID antigen standards are whole‐inactivated influenza virus and are quite different from the vaccine formulation.^20^, ^43^ Some studies have addressed this issue by preparing a working standard that is similar in form to the vaccines being tested,^41^, ^44^ but many unanswered questions remain such as the actual extent and impact of the perceived problem, and whether assay‐specific or product‐specific standards are needed. This is an example of a critical issue that may be best addressed with a future collaborative study involving the various interested parties. Any new approach to reagent preparation would need to be practical and coordinated with the WHO ERLs that are responsible for calibrating and distributing these reagents.

Collaborative studies, such as the ones in the early days of SRID evaluation and the more recent studies that have compared several of the alternative potency assays, are a valuable mechanism for advancing the field of alternative potency assay development and future studies will undoubtedly be crucial for resolving key issues that are common among the various assay approaches being pursued. Eventually, however, individual vaccine manufacturers will have to take the major step of implementation, and it may be that this will be easier for, or more attractive to, manufacturers of new products than for manufacturers of existing licensed vaccines. One possibly underappreciated impediment to continued progress may be the perception that an alternative potency assay must be all things to everyone and that manufacturers of all types of influenza vaccine products will be able to converge on a single type of assay. This may not be realistic for some of the reasons mentioned above, and there may yet be multiple potency assays in the future in spite of the clear advantages to a single accepted influenza potency that is suitable for all influenza vaccines.

The history of the SRID and its worldwide adoption is instructive, and a valuable, gratifying example of how the influenza community can work together to harmonise assays and testing for the common good. But, the success of that story can also mislead us into somewhat rigid thinking about how to approach the current situation. To quote the old aphorism (provenance unknown), “History does not repeat itself, but it often rhymes.”
